# Research on a Fiber Optic Oxygen Sensor Based on All-Phase Fast Fourier Transform (apFFT) Phase Detection

**DOI:** 10.3390/s22186753

**Published:** 2022-09-07

**Authors:** Pengkai Xia, Haiyang Zhou, Haozhe Sun, Qingfeng Sun, Rupert Griffiths

**Affiliations:** 1 School of Technology, Beijing Forestry University, Beijing 100083, China; 2 City and Urban Research Lab, LICA, Lancaster University, Lancaster LA1 4YW, UK

**Keywords:** fluorescence quenching principle, fiber optic oxygen sensor, all-phase fast Fourier transform algorithm

## Abstract

Fiber optic oxygen sensors based on fluorescence quenching play an important role in oxygen sensors. They have several advantages over other methods of oxygen sensing—they do not consume oxygen, have a short response time and are of high sensitivity. They are often used in special environments, such as hazardous environments and in vivo. In this paper, a new fiber optic oxygen sensor is introduced, which uses the all-phase fast Fourier transform (apFFT) algorithm, instead of the previous lock-in amplifier, for the phase detection of excitation light and fluorescence. The excitation and fluorescence frequency was 4 KHz, which was conducted between the oxygen-sensitive membrane and the photoelectric conversion module by the optical fiber and specially-designed optical path. The phase difference of the corresponding oxygen concentration was obtained by processing the corresponding electric signals of the excitation light and the fluorescence. At 0%, 5%, 15%, 21% and 50% oxygen concentrations, the experimental results showed that the apFFT had good linearity, precision and resolution—0.999°, 0.05° and 0.0001°, respectively—and the fiber optic oxygen sensor with apFFT had high stability. When the oxygen concentrations were 0%, 5%, 15%, 21% and 50%, the detection errors of the fiber optic oxygen sensor were 0.0447%, 0.1271%, 0.3801%, 1.3426% and 12.6316%, respectively. Therefore, the sensor that we designed has greater accuracy when measuring low oxygen concentrations, compared with high oxygen concentrations.

## 1. Introduction

Oxygen plays an irreplaceable role in both nature and human society. In recent years, various methods based on electrochemistry, chemical reactions, electromagnetism and optics have been developed to detect oxygen concentrations in gases or liquids. Among them, the fiber optic oxygen sensor based on the fluorescence burst principle has great superiority over other detection methods [1,2,3]. It has key advantages over other methods, such as: no oxygen consumption during detection; short response time; high sensitivity; high resolution; continuous determination of oxygen concentration in remote, hazardous or in vivo environments [4]; and oxygen concentration detection in both gases and liquids. Optical oxygen sensors break through the limitations of conventional Clark electrodes [5,6] and have been rapidly adopted for applications in chemical [7,8], clinical [9,10,11,12], microbiological [13] and marine detection [14] fields. In addition, the oxygen concentration of living plant tissue is an important index in the study of the physiological activities of plants and monitoring the growth and development of plants. An oxygen sensor that can detect the oxygen concentration of living plant tissue without affecting the normal growth and development of plants has great market value. It can mitigate the adverse effects on the production of agricultural and forestry crops caused by floods, field water and soil hardening, guide the design of artificial wetlands, and is of great significance for the production of agricultural and forestry crops and environmental protection [15,16,17,18].

In the study of fiber optic oxygen sensors, phase demodulation is an important factor that affects detection results. Dongqiu Zhou et al. used a lock-in amplifier SR830 for phase demodulation in fiber optic oxygen sensors [19], achieving high detection accuracy; however, the SR830 was a laboratory instrument, which was costly, large and not easily portable, and it could not achieve oxygen concentration detection in complex environments. Fupeng Wang et al. applied a lock-in amplifier with AD630 as the phase demodulation core to fiber optic oxygen sensors [20], which was small, portable and robust; however, a low-pass filter was often added after the AD630, which made the system noisy and of low accuracy, and a 160,000 times averaging algorithm was required for better results. Xiangdong Huang et al. proposed the all-phase fast Fourier transform (apFFT) [21] based on the fast Fourier transform (FFT), which had the advantages of suppressing spectral leakage and phase invariance.

In our study, the apFFT was applied to the fiber optic oxygen sensor. First, the excitation photoelectric signal and the fluorescent electric signal were sampled. The sampled signals were then processed by the apFFT and arc tangent transform, and the result obtained was two phases. Corresponding processing was performed according to the sign of the real and imaginary parts of the phase, and the two processed phases were subtracted to finally obtain a difference. The phase difference was the hysteresis phase shift of the fluorescence relative to the excitation light, which could be converted into the corresponding oxygen concentration. This method can effectively reduce the size of the fiber optic oxygen sensor, reduce costs and overcome the problems of high noise and low accuracy of the traditional analog lock-in amplifier.

## 2. Principle

### 2.1. Fiber Optic Oxygen Sensor

When the excitation light of a specific wavelength band is conducted to the fluorescent material molecules, it will cause electrons to undergo an energy state jump from the ground-state energy level to the excited-state energy level; this is known as the excitation of luminescent material [22]. The excited-state molecule is unstable, and it will release the energy of the electrons in the higher energy level by emitting photons, after which the electrons drop to the lowest vibration energy level of the first excited state, resulting in the fluorescence phenomenon; this process is called photoluminescence [23].

When a fluorescent burster is present in the environment, new photophysical and photochemical deactivation pathways are introduced. The process of interaction between the excited- or ground-state molecules of the fluorescent substance and the molecules of the fluorescent burster (i.e., oxygen molecules), which results in a transfer of fluorescence energy and a decrease in fluorescence efficiency, is known as fluorescence quenching [24,25,26,27,28].

The fluorescence quenching process competes with the photoluminescence process, resulting in a decrease in the intensity of the fluorescence produced by radiation and a shortening of the lifetime of the excited state of the fluorescent molecule. Both fluorescence intensity and fluorescence lifetime vary with the concentration of fluorescent quenching agent, and the relationship is described by the Stern–Volmer equation [29,30,31,32,33,34,35].

(1) $$\frac{I_{0}}{I}=\frac{\tau_{0}}{\tau }=1+K\left[Q\right]$$

where $I_{0}$ is the fluorescence intensity under anaerobic conditions, I is the fluorescence intensity under aerobic conditions, $\tau_{0}$ is the fluorescence lifetime under anaerobic conditions, τ is the fluorescence lifetime under aerobic conditions, K is the Stern–Volmer constant, and Q is the oxygen concentration.

A greater concentration of gaseous oxygen and dissolved oxygen results in a lower intensity and shorter lifetime of fluorescence due to the quenching effect of oxygen molecules; consequently, the corresponding amplitude of the output fluorescence signal is smaller, and there is a smaller lag phase shift with the excitation light signal. Therefore, the oxygen concentration can be detected by fluorescence intensity or fluorescence lifetime. However, fluorescence intensity is susceptible to external factors during transmission, while fluorescence lifetime is an intrinsic parameter of the substance, which is not subject to changes in external factors and has good anti-interference ability. Therefore, the detection of oxygen concentration by fluorescence lifetime is more accurate.

Compared with measuring fluorescence intensity, it is more difficult to measure the fluorescence lifetime directly, but when the excitation light frequency is fixed, the fluorescence lifetime is proportional to the tangent of the fluorescence lagging the phase shift of the excitation light [35], as shown in the following equation.

**Figure 1:**
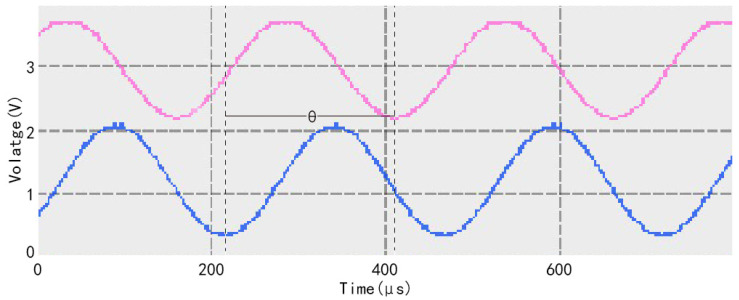
Phase relationship between fluorescence generated by oxygen-sensitive membrane and excitation light under laser irradiation.

(2) tanθ=ωτ

where ***θ*** is the phase shift of fluorescence lagging behind the excitation light, ***ω*** is the frequency of the excitation light, and ***τ*** is the fluorescence lifetime. The detection of oxygen concentration can be accomplished by the phase shift of fluorescence lagging behind the excitation light. Figure 1 shows the phase relationship between the fluorescence generated by the oxygen-sensitive membrane and the excitation light under the excitation light irradiation. In the figure, the blue sine wave is the excitation light signal, the pink sine wave is the fluorescence signal, and ***θ*** is the lagged phase shift of the fluorescence signal with respect to the excitation light signal [36].

### 2.2. All-Phase Fast Fourier Transform (apFFT)

According to the Shannon sampling theorem [37,38], an analog signal can be recovered without distortion when the sampling frequency is greater than twice the highest frequency in the analog signal spectrum. The Fourier transform can be used to expand periodic signals into trigonometric functions. In practical applications, the FFT can be used to obtain the phase information of sinusoidal signals at different frequencies of the constituent original signals, while the noise and spectral leakage problems of the FFT can affect the accuracy of phase demodulation.

The apFFT algorithm was proposed by Professors Zhaohua Wang and Zhengxin Hou of Tianjin University and has characteristics of constant initial phase and effective prevention of spectrum leakage. The traditional method is to take the n data obtained from sampling by FFT and the amplitude or the square of the amplitude before output. The full-phase analysis method takes into account only one of the input data segmentation cases, and if all possible segmentations of the input data are taken into account and the phases are compensated by each other, then the performance of the spectrum analysis will be significantly improved [39,40]. This is because FFT is used to obtain an infinitely long sequence by making a period extension of the truncated sequence. When not sampled at equal intervals, after doing the period stretching, there will be a jump in the signal at the beginning and end, which is not consistent with the original signal, and the analysis of the spectrum will exhibit frequency leakage [41]. The apFFT spectral analysis method has excellent frequency leakage resistance and phase invariance, and is suitable for spectral analysis that contains multiple dense frequency components [42]. The difference between the apFFT algorithm and other FFTs lies in the data preprocessing. The data preprocessing of the apFFT algorithm is relatively complex and includes two parts: data preprocessing and the FFT algorithm.

Therefore, in this paper, we propose a method to reduce the effect of noise interference and suppress spectral leakage by adding full-phase preprocessing before the FFT.

**Figure 2:**
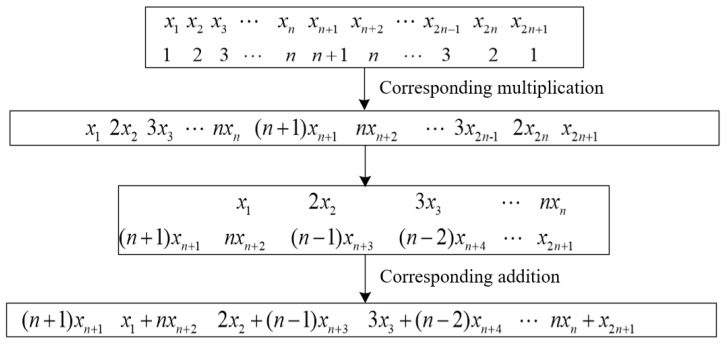
Full-phase preprocessing process.

Full-phase preprocessing is a convolutional window added before FFT processing with n points, and the array with 2n + 1 elements $\left\{x_{1},x_{2},\ldots ,x_{n},\ldots ,x_{2n},x_{2n+1}\right\}$ is weighted and phase-shifted to obtain an array with n elements $\left\{y_{1},y_{2},\ldots ,y_{n}\right\}$; the process is shown in Figure 2.

The expression is:

(3) $$y_{1}=(n+1)x_{n+1}$$

(4) $$y_{i}=(i-1)x_{i-1}+(n+2-i)x_{n+i},\;(i=2,3,\ldots ,n)$$

The lag phase shift of the fluorescence with respect to the excitation light is obtained by performing an FFT on the array obtained after adding windows and an inverse tangent transformation on the real and imaginary parts of the corresponding frequencies after processing.

## 3. System Design

### 3.1. System Structure of Fiber Optic Oxygen Sensor

**Figure 3:**
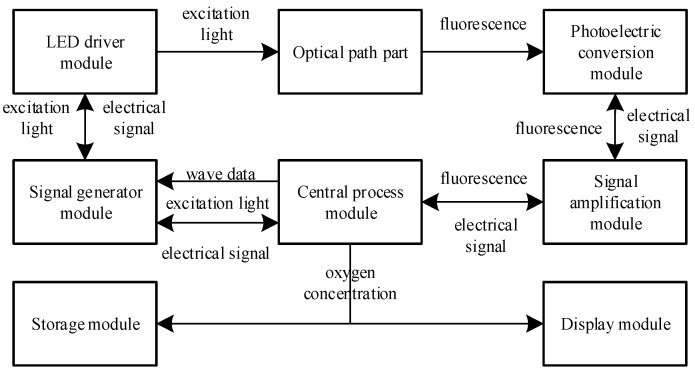
System block diagram of fiber optic oxygen sensor.

The fiber optic oxygen sensor designed in this paper consisted mainly of a central processing module, a signal generation module, a light-emitting diode (LED) driver module, an optical path component, a photoelectric conversion module, a signal amplification module, a display module and a storage module. Its system block diagram is shown in Figure 3.

The central processing module employed an STM32F103 microcontroller (STMicroelectronics, Geneva, Switzerland) with 32-bit Cortex-M3 core, which sent the sinusoidal waveform data of the excitation light to the signal generation module and sampled the output sinusoidal waveform of the signal generation module. The signal generation module adopted the direct digital frequency synthesis chip AD9833 (Analog Devices, Norwood, MA, USA), which amplified the output sine wave using the operational amplifier AD823 (Analog Devices, Norwood, MA, USA) and then input it into the LED driver module as the excitation photoelectric signal, and controlled the LED through the operational amplifier LTC6256 (Analog Devices, Norwood, MA, USA) and transistor BC847BW (Nexperia, Nijmegen, The Netherlands) to generate the excitation light, with the intensity varying according to the sine wave.

The generated excitation light was transmitted to the oxygen-sensitive layer via the beamsplitter, glass bulb and optical fiber in the optical path section. At the oxygen-sensitive layer, the fluorescent material ground-state molecules absorbed the energy of the excitation light, photoluminescence occurred, and fluorescence was generated. At the same time, the fluorescent material excited-state molecules and ground-state molecules interacted with oxygen molecules, and fluorescence quenching occurred, resulting in a reduction in fluorescence intensity and shortened fluorescence lifetime. The photoluminescence of fluorescence and the fluorescence burst that shortened the fluorescence lifetime competed with each other to produce fluorescence that lagged behind the excitation light by a certain phase shift. The fluorescence was transmitted back to the photoelectric conversion module through the optical fiber, glass beads, beamsplitter and filter in the optical path section, and was converted into an electrical signal by the silicon photocell BPW34S (Vishay, Malvern, PA, USA). Due to the small amplitude of the electrical signal output from the photoelectric conversion module and the poor driving capability, it required amplification by the multi-channel transimpedance amplifier MTI04CS (MAZeT Gmbh, Austria, Germany) of the signal amplification module before passing to the central processing module for sampling. The central processing module processed the excitation photoelectric signal and the fluorescent electric signal to obtain the oxygen concentration, and transmitted it to the storage module and the display module.

**Figure 4:**
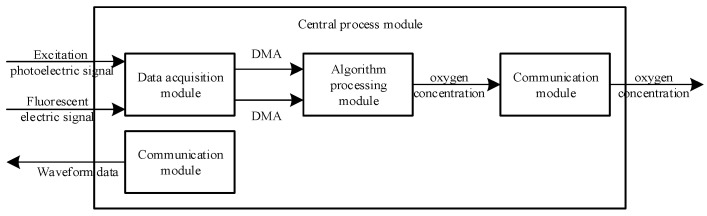
Internal structure diagram of the central processing module.

The central processing module included the data acquisition module, algorithm processing module and communication module. Its internal structure block diagram is shown in Figure 4.

The excitation photoelectric signal and fluorescence electric signal were collected by the data acquisition module and then transmitted by direct memory access (DMA) to the algorithm processing module. In the algorithm processing module, two arrays containing 2048 elements, each transmitted by DMA, were pre-processed by full phase to obtain two arrays, each of 1024 elements. The FFT outputted the real and imaginary parts of the sinusoidal waveforms of each frequency that formed the excitation photoelectric signal and the fluorescent electric signal. The real and imaginary parts of the corresponding frequencies were selected, and the lag phase shift of the fluorescence with respect to the excitation light was obtained by performing an inverse tangent transform on them. Based on the relationship between the lag phase shift of fluorescence with respect to the excitation light and the fluorescence lifetime, and the relationship between the fluorescence lifetime and the oxygen concentration, the lag phase shift of fluorescence with respect to the excitation light was converted to the oxygen concentration and transmitted by the communication module. Meanwhile, the communication module that sent waveform data to the signal generation module operated independently.

In summary, this section introduces in detail the various parts of the fiber optic oxygen sensor designed by us, including the central processing module, signal generation module, optical path component and photoelectric conversion module. In this study, the system modules involved in the fiber optic oxygen sensor were integrated into the printed circuit board, which not only ensures volume, but also enables better packaging, which is convenient for long-term field measurement and use. The main system modules involved in the sensor are shown in Table 1.

### 3.2. The Optical Path Component of the Fiber Optic Oxygen Sensor

**Figure 5:**
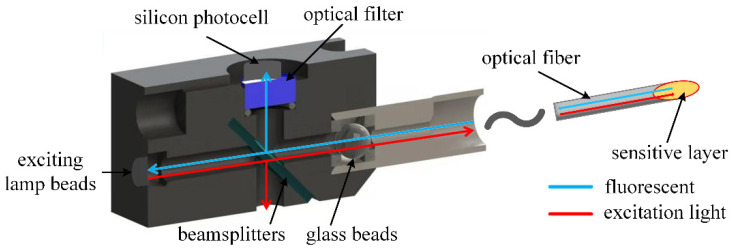
Schematic diagram of the structure of the optical path component.

The optical path component included the exciting lamp beads, beamsplitter, glass ball beads, filter, optical fiber and oxygen-sensitive layer. To prevent the influence of external light sources, a mold was created to separate the optical path, including the optical fiber, from the outside world. The optical fiber and the mold were connected through a plug, and the oxygen-sensitive layer was connected to the end of the optical fiber. The structure schematic diagram is shown in Figure 5.

In this paper, OXROB10 fiber optic oxygen probes (Pyroscience, Aachen, Germany) were used. The OXROB10 probes were coated with a REDFLASH indicator at the end of the fiber as an oxygen-sensitive film. Multimode optical fiber was used. The REDFLASH indicator required a red light excitation of 610–630 nm. The fluorescence band was 760–790 nm.

Since the excitation spectrum of the oxygen-sensitive film was required to be 610–630 nm (within the red spectrum range), a light source within the spectrum range of the generated spectrum should be selected. The light source selected in this paper was 3528 lamp beads (TAIHONGLED, Dongguan, China), which can produce 620–625 nm red light, meeting the spectrum requirements of oxygen-sensitive film. Since the lamp bead was packaged as a ball head, it could also focus the light and reduce the loss of excitation light.

**Figure 6:**
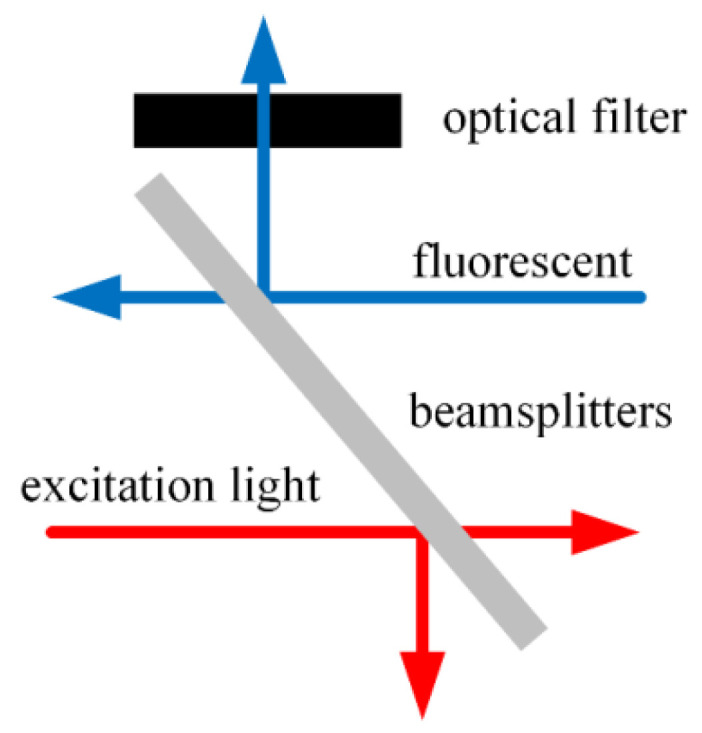
Schematic diagram of cross section of conductive optical path.

A cutoff beamsplitter (Model: HB720, Zhenhua Optoelectronics, Nantong, China) with 50% transmission and 50% reflection was used to separate the excitation light and fluorescence, and a filter was added between the beamsplitter and the circuit for filtering treatment. The cross-section diagram of the conduction light path is shown in Figure 6.

Placing the beam splitter at an angle of 45° with the excitation light caused the excitation light transmitted through the beam splitter into the optical fiber to account for 50% of the excitation light generated by the light source, and the fluorescence reflected by the beam splitter into the circuit accounted for 50% of the fluorescence from the oxygen-sensitive film into the optical fiber. A transparent glass ball was added between the conductive optical path and the optical fiber of the optical fiber probe to condense the excitation light so that as much of it entered the optical fiber as possible. To reduce the interference of fluorescence detection, the cutoff beamsplitter HB720 was placed between the spectroscope and the circuit to filter the excitation light and external stray light before the fluorescence was converted into an electric signal.

The optical path was as follows: the excitation light generated by the excitation lamp beads passed through the beamsplitter with a splitting ratio of 3:7 and entered the optical fiber after being concentrated by the glass ball beads, and was then conducted to the oxygen-sensitive layer at the end of the optical fiber. At the oxygen-sensitive layer, the fluorescent material ground-state molecules absorbed the excitation light to reach the excited state and then deactivated back to the ground state, releasing energy to produce fluorescence. At the same time, in an oxygen environment, the excited state molecules of the fluorescent substance or the ground state molecules interacted with oxygen molecules to transfer the energy brought by the excitation light, which decreased the fluorescence intensity and shortened the fluorescence lifetime. Photoluminescence and the fluorescence burst competed with each other—photoluminescence produced fluorescence and the fluorescence burst shortened the fluorescence lifetime—and produced fluorescence corresponding to the fluorescence lifetime under the oxygen concentration of the environment. Since the fluorescence lifetime was proportional to the tangent of the lag phase shift of the fluorescence with respect to the excitation light, different concentrations of oxygen environments corresponded to different lag phase shifts of the fluorescence with respect to the excitation light. The fluorescence was transmitted back along the optical fiber, passed through the glass spherical beads and was then reflected by a beamsplitter and filtered by a light filter before entering the silicon photocell for photoelectric conversion.

## 4. Results and Discussion

### 4.1. Experimental Results of apFFT

Before applying the apFFT to the fiber optic oxygen sensor, a test experiment was designed to test the phase detection performance of the transform. The control module and power conversion module in the sensor circuit were taken out separately, and the power conversion module was used to supply power to the control module and provide the analog-to-digital converter (ADC) reference voltage. The software system only needed to implement the data acquisition module and algorithm processing module, remove the phase difference and oxygen concentration conversion sub-module in the algorithm processing module, subtract the phase of the algorithm processing module after arctangent transformation, average the 100 consecutive differences, and output the average value as the result. The common source signal generator DG1022U (RIGOL, Suzhou, China) was used to generate two sine waves with a frequency of 4 KHz instead of the excitation photoelectric signal and fluorescent electric signal as the measured input signals, which were connected to the corresponding pins of the two ADC sampling channels of the control module. By adjusting the phase difference of the output sine wave of the signal generator, the lag phase shift of fluorescence relative to excitation in different oxygen concentration environments was simulated.

The system first samples two input sine waves at a sampling frequency of 16 KHz. Since the number of points selected for the fast Fourier transform was 1024, and the all-phase preprocessing would halve the sampling sequence, it was necessary to sample 2048 points for each of the two sine waves. Two p-sequences of 1024 points were obtained by the all-phase preprocessing of two sampling sequences of 2048 points. The two p-sequences were subjected to the fast Fourier transform of 1024 points, and the transform results were the real and imaginary parts of the corresponding frequency components, increasing from 0 Hz to 15.625 Hz. The 257th data point was the real part and the imaginary part of the 4 KHz sine wave. The phase of the sine wave was obtained by arctangent transformation of the quotient of the imaginary part and the real part. The phase difference of two sine waves was obtained by subtracting the phases of two sine waves. To reduce the error, the average value was taken for every 100 phase differences, and the average value was taken as the detection result.

**Figure 7:**
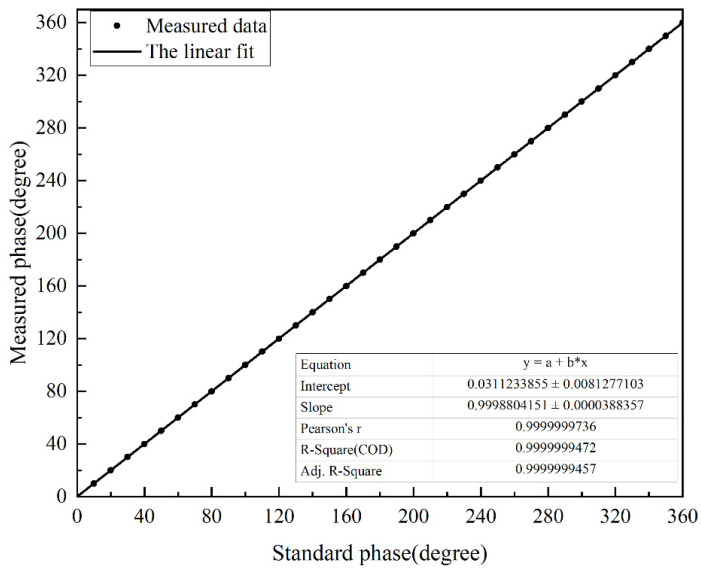
Fitted curve of apFFT measurement points.

The phase difference between the two sine waves increased from 0° to 360° in units of 10°. The apFFT was used to detect them, and fit the detection results. The fitting curve is shown in Figure 7.

**Figure 8:**
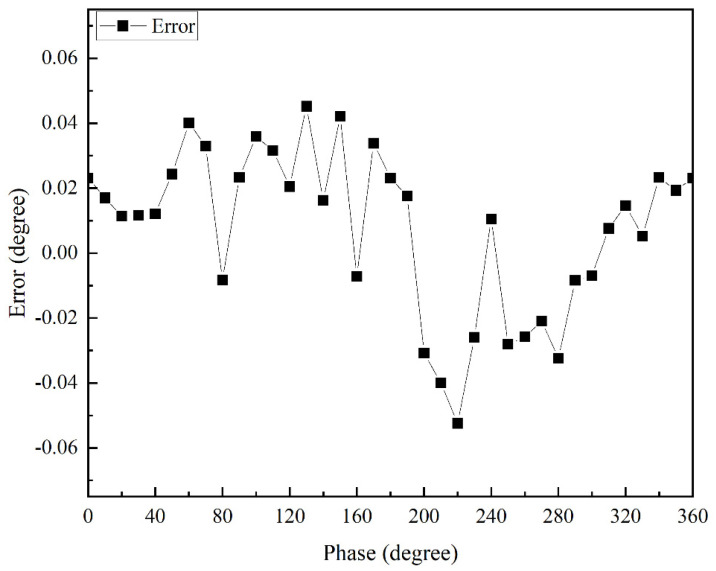
Accuracy of apFFT.

It can be seen that the goodness of fit of its fitted curve was very good, and the linearity reached 0.9999999457. The experimentally measured response time was less than 0.2 s, and the resolution reached 0.0001°. The accuracy of the apFFT is shown in Figure 8, which reached 0.05°.

**Figure 9:**
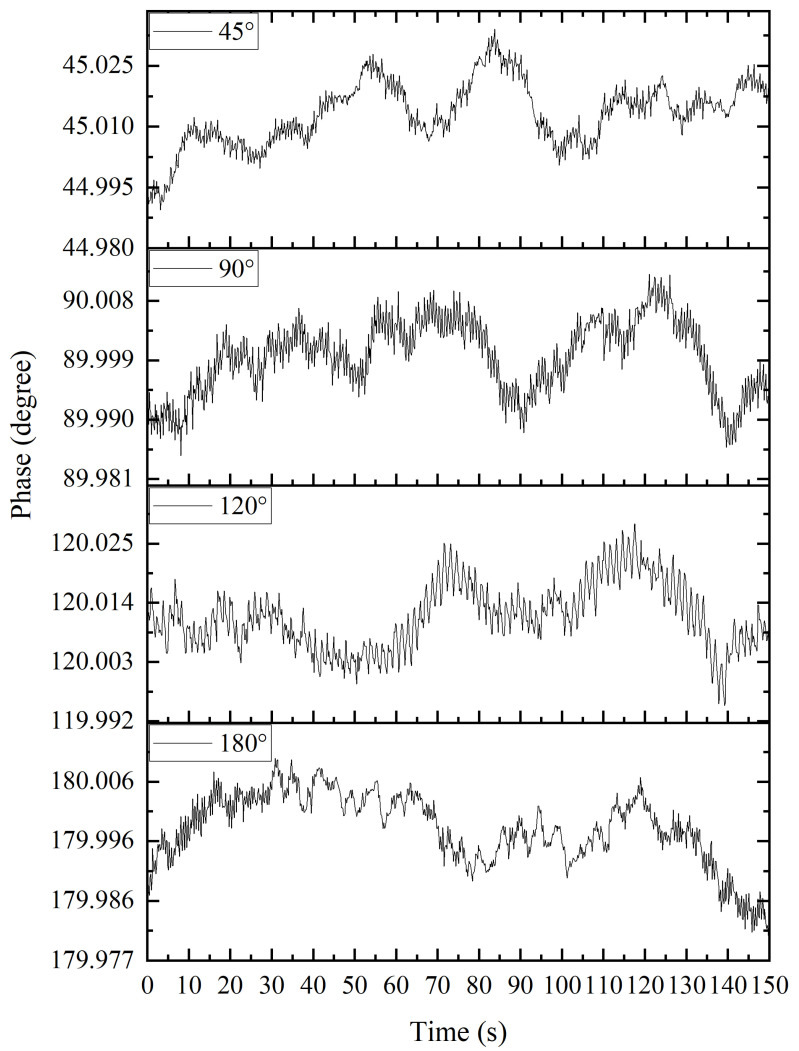
Measurement results of 45°, 90°, 120° and 180° phase differences using apFFT.

The accuracy of the apFFT is shown in Figure 9. The measurement results of 45°, 90°, 120° and 180° phase differences using the apFFT transform were within 0.01° of noise, and the phase shift change range was within 0.05° for long-term measurements. Meanwhile, the apFFT was used to effectively circumvent the shortcomings of the lock-in amplifier AD630 with its high noise level, while its stability was good.

### 4.2. Experimental Results of Fiber Optic Oxygen Sensor

**Figure 10:**
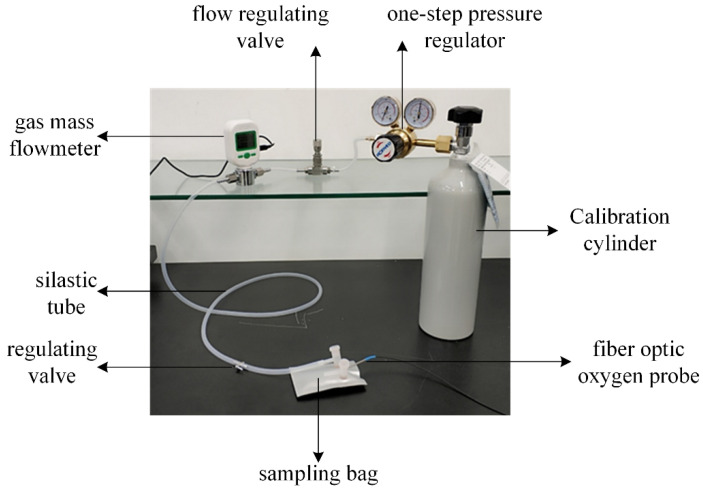
Air connection diagram.

For the fiber optic oxygen sensor experiment, a gas cylinder filled with a fixed concentration of oxygen was used as the gas source (the accuracy of gas concentration was greater than 99.999%), and the gas cylinder was connected to the inlet of a single-stage pressure regulator, through which the pressure of the gas outlet from the cylinder was controlled. The gas outlet of the single-stage pressure regulator was connected to the gas inlet of the flow regulator valve through a silicone tube, and the flow rate of the gas outlet was controlled through the flow regulator valve and displayed through a gas mass flow meter. Finally, the gas flow was controlled by the gas volume regulating valve to switch the gas circuit and pass the gas into the gas sampling bag. When detecting oxygen concentration, the blowing method was used. After the gas sampling bag was full of gas, the outlet of the gas sampling bag was opened, while the gas cylinder continued to inflate the gas sampling bag so that the gas in the gas sampling bag could flow out. The fiber optic oxygen sensor was inserted into the gas sampling bag to detect the oxygen concentration in the gas sampling bag. The gas circuit connection is shown in Figure 10.

**Figure 11:**
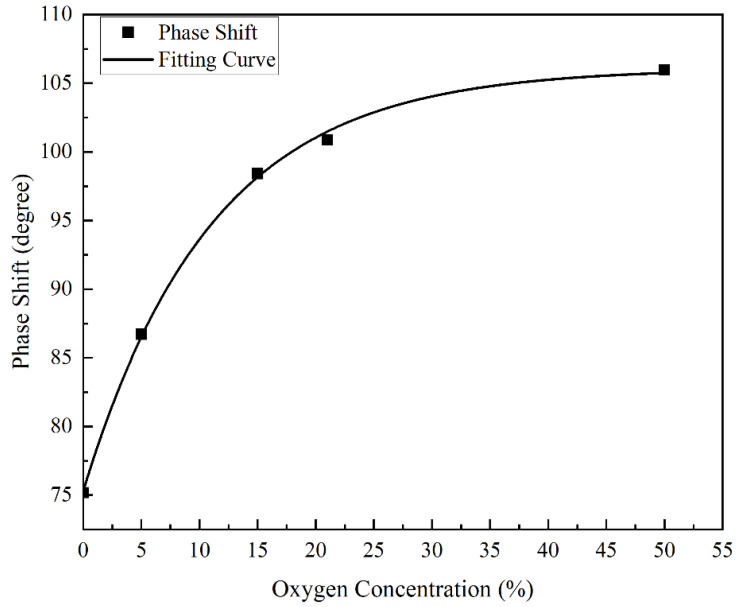
Lag phase shift of fluorescence relative to excitation for different concentrations of oxygen.

The lag phase shifts of the fluorescence relative to the excitation light of the fiber optic oxygen sensor were determined for 0%, 5%, 15%, 21% (air) and 50% concentrations of oxygen and nitrogen gas mixtures, as shown in Figure 11. As described above for the photoluminescence and fluorescence burst mechanism, the lag phase shift of fluorescence with respect to excitation light increased as the oxygen concentration increased. Between 0% and 50% oxygen concentrations, the lag phase shift of fluorescence with respect to the excitation light, increased by about 30°. At low oxygen concentrations, the phase shift changed significantly; as the oxygen concentration increased, the increment of the phase shift became smaller.

To convert the fluorescence to the oxygen concentration in the environment after the lagging phase shift of the fluorescence, relative to the excitation light detected by the fiber optic oxygen sensor, it was necessary to fit the curve according to the relationship between the oxygen concentration and the lagging phase shift of the fluorescence relative to the excitation light, as shown in Figure 11. It can be seen from the figure that when the oxygen concentration in the measured environment increased, the lagging phase shift of fluorescence relative to the excitation light increased, but the slope decreased, which conformed to the basic principle of the fluorescence quenching mechanism and phase shift method. The figure also shows that as the oxygen concentration in the measured environment increased, the detection error of the fiber optic oxygen sensor continued to increase. In the environment with an oxygen concentration of 0%, the detection error of the fiber optic oxygen sensor was 0.0447%; in the environment with an oxygen concentration of 5%, the detection error of the fiber optic oxygen sensor was 0.1271%; in the environment with an oxygen concentration of 15%, the fiber optic oxygen sensor detection error was 0.3801%; in the environment with an oxygen concentration of 21%, the detection error of the fiber optic oxygen sensor was 1.3426%; and in the environment with an oxygen concentration of 50%, the detection error of the fiber optic oxygen sensor reached 12.6316%. Therefore, the fiber optic oxygen sensor was not suitable for the detection of high oxygen concentrations in the environment, and the detection results were more accurate in environments with lower oxygen concentrations. The fitted curve equation in Figure 11 is:

(5) $$y=a+b\times \exp\left(-\frac{x}{t}\right)$$

where *a* = 106.0793, *b* = −30.76477, *t* = 11.06124, *x* is the oxygen concentration in the environment, and *y* is the lagging phase shift of fluorescence relative to the excitation light. Since the fiber optic oxygen sensor detected the lagging phase shift of fluorescence relative to the excitation light, the oxygen concentration in the environment needed to be derived from the lagging phase shift. Therefore, the lagging phase shift of fluorescence relative to the excitation light should be changed to an independent variable, and the oxygen concentration in the environment should be changed to a dependent variable. The fitting curve equation is as follows:

(6) $$y=-t\times \ln\left(\frac{x-a}{b}\right)$$

where *a* = 106.0793, *b* = −30.76477, *t* = 11.06124, *x* is the lagging phase shift of fluorescence relative to the excitation light, and *y* is the oxygen concentration in the environment.

**Figure 12:**
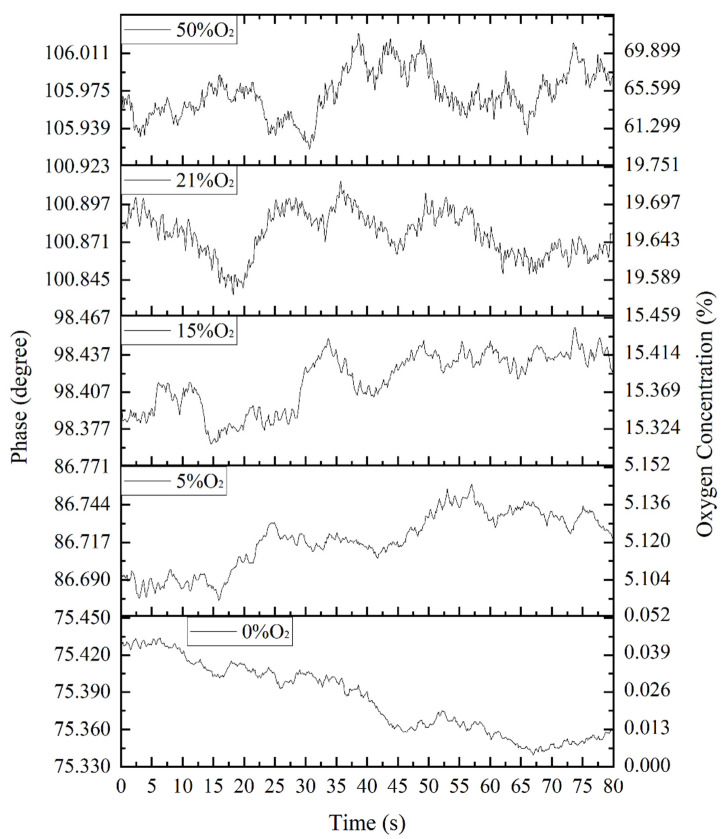
The results of multiple detections by the sensor under various oxygen concentrations.

The fiber optic oxygen sensor was tested for stability at 0%, 5%, 15%, 21% (air) and 50% concentrations of oxygen in an oxygen–nitrogen gas mixture. We measured the sensor at five concentrations, and showed that the phase shift varied within 0.1° when measured at the same oxygen concentration for a long time. The phase-difference data above was converted into oxygen concentration according to the fitting curve, and the results are shown in Figure 12.

Regarding noise, when the concentration of oxygen in the environment was 0%, the noise was 0.0340%; when the concentration of oxygen in the environment was 5%, the noise was 0.0476%; when the concentration of oxygen in the environment was 15%, the noise was 0.1363%; when the concentration of oxygen in the environment was 21%, the noise was 0.1660%; and when the concentration of oxygen in the environment was 50%, the noise was 13.0455%. It can be seen that when the oxygen concentration was between 21% and 50%, the impact of noise increased sharply. When the oxygen concentration in the environment was low, the stability of the fiber optic oxygen sensor was good; when the oxygen concentration in the environment was high, the stability of the fiber optic oxygen sensor was poor.

## 5. Conclusions

This paper proposes a fiber optic oxygen sensor based on the apFFT. It applied the apFFT to the phase detection of the fiber optic oxygen sensor and tested the performance of the apFFT algorithm implemented by STM32F103ZET6 and the developed fiber optic oxygen sensor. The experimental results showed that the apFFT had good performance regarding phase detection, and the coefficient of determination of the fitted curve reached 0.9999999457 in the phase detection from 0 to 360°, and the accuracy reached 0.05°. Meanwhile, the apFFT effectively avoided the problem of noise caused by use of the lock-in amplifier AD630, and had good stability, with its noise within 0.01° and the phase shift change range within 0.05° during long-term measurement. The noise of the fiber optic oxygen sensor using apFFT was significantly reduced, and its phase shift change range for long-term measurements was within 0.1°. The fiber optic oxygen sensor based on apFFT described in this paper is low cost, does not require a device for phase detection and can detect the fluorescence intensity by the square root mean value of the real and imaginary parts after FFT while detecting the phase. The fiber optic oxygen sensor is small, easy to carry and can be used in the field for the long-term detection of oxygen concentrations in gaseous or liquid environments.

## Figures and Tables

**Table 1:** The main system modules involved in the sensor.

System Elements	Composition
Central processing module	Data acquisition module
	Algorithm processing module
	Communication module
Signal generation module	AD9833 chip
LED driver module	3528 lamp beads
	LTC6256 operational amplifier
	BC847BW transistor
Optical path component	Beamsplitter
	Glass ball beads
	Filter
	Optical fiber
	Oxygen-sensitive layer
Photoelectric conversion module	Photoresist and photodiode
Signal amplification module	MTI04CS operational amplifier

## Data Availability

Not applicable.
